# The Diagnostic Dilemma in Delayed Subarachnoid Hemorrhage: A Case Report

**DOI:** 10.5811/cpcem.1586

**Published:** 2023-08-02

**Authors:** Danielle A. Bazer, Nicholas Koroneos, Matthew Orwitz, Jordan Amar, Ryan Corn, Elizabeth Wirkowski

**Affiliations:** * Stony Brook University Hospital, Department of Neurology, Stony Brook, New York; † Johns Hopkins Medicine, Department of Neurology, Baltimore, Maryland; ‡ Washington University in St. Louis, Department of Neurology, St. Louis, Missouri

**Keywords:** subarachnoid hemorrhage, lumbar puncture, radiologically negative

## Abstract

**Introduction:**

Radiologically negative subarachnoid hemorrhage (SAH) has a low incidence and is associated with good clinical outcomes.

**Case Report:**

We present the case of a 44-year-old male with new-onset headaches, which began one week prior while bike riding. At an outside hospital, he had normal computed tomography head and angiogram. He declined a lumbar puncture. Over the following week, the headache was persistent. He lacked meningeal signs. Repeat studies were normal. Lumbar puncture was positive for xanthochromia.

**Conclusion:**

Radiologically negative SAH should be included in the differential diagnosis of patients presenting with unremitting headache in the setting of recent exercise, despite negative imaging, and meningeal signs.

## INTRODUCTION

The unifying features of all types of subarachnoid hemorrhage (SAH) are an average age of onset at 55 years old with a male predominance (despite aneurysmal SAH having a female predominance).^1^,^2^ Patients with an idiopathic etiology tend to have a less severe disease course with fewer complications.^1^ However, 85% of SAH are secondary to aneurysmal rupture.^3^ Vascular imaging is suggested after the diagnosis of SAH is made.^3^ Although at a low incidence, less than 1% of cases are diagnosed by spectrophotometric detection of a bilirubin peak in the cerebrospinal fluid (CSF) obtained from a lumbar puncture (LP), as reflected in this case.^4^–^6^

## CASE REPORT

This is a case of a 44-year-old male, without past medical history, who initially presented due to new-onset headaches. The patient reported no personal or family history of headaches. His symptoms began one week prior to presentation while exercising vigorously on his exercise bike. During the last few minutes of his workout, he developed an acute onset, band-like headache with radiation to his cervical spine. He first went to a local emergency department where he underwent CT head and CT angiogram, which were reportedly normal. He was offered an LP, but he declined.

Over the following week, the headache was persistent, with new-onset tinnitus, intermittent phonophobia, and continued pain radiation down his spine to his central lower back. He denied pain with eye movements, photophobia, nausea, vomiting, and positional changes. His neurological exam was unremarkable, with absent meningeal signs. Head CT, magnetic resonance imaging (MRI) of the brain (Image), cervical, thoracic, and lumbar spine were normal. Magnetic resonance angiogram of the head and neck was also normal. Complete blood count, chemistry, coagulation studies, erythrocyte sedimentation rate, and C-reactive protein were within normal limits.

An LP revealed yellow-colored CSF positive for xanthochromia, 223 white blood cells (97% mononuclear cells), and 6,000 red blood cells. Protein and glucose levels were normal. He subsequently underwent diagnostic cerebral angiogram, which was normal. The patient was diagnosed with a radiographic-negative SAH and was treated with a trial of steroids upon discharge home. He was followed up outpatient at two weeks and six weeks from discharge. By six-week follow-up, he reported 99% improvement of his symptoms.

## DISCUSSION

In 85% of SAH cases, aneurysms are the cause. Non-aneurysmal SAH is defined as SAH without vascular lesions on angiography and can be further divided into perimesencephalic and non-perimesencephalic non-aneurysmal SAH. Aneurysmal SAH has a vascular lesion.^7^ Although non-contrast CT of the brain is the preferred initial imaging modality, CT angiogram, diagnostic angiogram, and MRI can aid in the diagnosis to further understand cerebrovascular anatomy and aneurysms.^8^ The sensitivity of MRI in SAH ranges from 50–94% with an acute SAH and 33–100% in subacute SAH. The most sensitive sequence is T2 with a sensitivity of 94%. The specificity is 98.5%.^9^ Lu et al found that digital subtraction angiogram had a sensitivity of 91.3% of detecting aneurysms less than 3 millimeters (mm); 94.0% for aneurysms between 3 mm but <5 mm; 98.4% for aneurysms between 5 mm and <10 mm; and 100% for aneurysms ≥10 mm.^10^

This case highlights the importance of the above evaluation with regard to sentinel headaches. Sentinel headaches signify a particularly severe headache that precedes a second episode of a profound headache secondary to intracranial aneurysm rupture.^11^ Typically, sentinel bleeds are believed to be a warning as an aneurysm starts to bleed and will subsequently rebleed a few days later, necessitating medical attention and evaluation.^11^ Gambhir et al in 2009 cited that 38% of patients with aneurysmal SAH had sentinel headaches, with misdiagnosis being a key problem.^12^

**CPC-EM Capsule**

What do we already know about this clinical entity?

*Although rare, we know that subarachnoid hemorrhages can be radiologically negative.*

What makes this presentation of disease reportable?

*Although the patient lacked meningeal signs and had negative neuro-imaging on two occasions, the lumbar puncture confirmed the diagnosis of radiologically negative subarachnoid hemorrhage.*

What is the major learning point?

*It is imperative to obtain a lumbar puncture when radiographic studies are negative to rule out subarachnoid hemorrhage as the etiology of a headache.*

How might this improve emergency medicine practice?

*This case will likely urge emergency medicine providers to obtain lumbar punctures on patients who have an unremitting headache and negative neuro-imaging.*

As a sentinel headache typically does not share the cardinal features of spontaneous SAH, such as focal neurological deficits or nuchal rigidity, it is common for sentinel headaches to be misdiagnosed as a different headache type.^13^,^14^ Therefore, in individuals who are not prone to having headaches a complete evaluation, including imaging and LP, should be obtained.^13^ For our patient, it was key to have a LP performed, as he had both CT- and MRI-negative brain and vessel imaging on two separate occasions, as well as an unrevealing diagnostic angiogram. Given the persistence of bilirubin and xanthochromia in the spinal fluid up to 15 days after hemorrhage, it is imperative to obtain a LP when the radiographic studies are negative to elucidate whether SAH is the etiology of the headache.^15^

## CONCLUSION

This case highlights the importance of including radiologically negative SAH in the differential diagnosis of patients presenting with unremitting headache in the setting of recent exercise, despite negative imaging and the absence of meningeal signs on physical exam.

## Figures and Tables

**Image:**
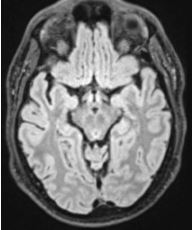
This is a normal magnetic resonance image of the brain. There is no blood in the perimesencephalic area.
